# The Influence of COVID-19 Pandemic Lockdown on the Physical Performance of Professional Soccer Players: An Example of German and Polish Leagues

**DOI:** 10.3390/ijerph18168796

**Published:** 2021-08-20

**Authors:** Łukasz Radzimiński, Alexis Padrón-Cabo, Marek Konefał, Paweł Chmura, Andrzej Szwarc, Zbigniew Jastrzębski

**Affiliations:** 1 Department of Physiology and Biochemistry, Gdansk University of Physical Education and Sport, 80-336 Gdansk, Poland; zb.jastrzebski@op.pl; 2 Department of Physical Education and Sport Science, University of A Coruña, 15071 A Coruña, Spain; a.cabo@udc.es; 3 Department of Biological and Motor Sport Bases, University School of Physical Education, 51-612 Wroclaw, Poland; marek.konefal@awf.wroc.pl; 4 Department of Team Games, University School of Physical Education, 51-612 Wroclaw, Poland; pawel.chmura@awf.wroc.pl; 5 Department of Team Sports, Gdansk University of Physical Education and Sport, 80-336 Gdansk, Poland; andrzej.szwarc@awf.gda.pl

**Keywords:** football, match running performance, time-motion analysis, Bundesliga, Ekstraklasa

## Abstract

The purpose of this study was to investigate whether the in-season pandemic lockdown influenced physical match performance in professional soccer players who participated in the competition of German Bundesliga and Polish Ekstraklasa. The data from 306 games for German Bundesliga and 296 games for Polish Ekstraklasa were divided into before- and after-lockdown periods. The physical performance of German Bundesliga teams after the 63-day COVID-19 lockdown did not differ significantly from that in the first part of the season. In contrast, Polish Ekstraklasa teams, after the 81-day lockdown, displayed significantly shorter total distances (*p* < 0.001), shorter high-intensity running distances (*p* = 0.03), and fewer high-intensity actions (*p* = 0.02). Moreover, when the effective playing time was considered, teams covered significantly less relative total distance (*p* < 0.001) and relative high-intensity running distance (*p* = 0.02). The results of the current study suggest that physical performance during the matches of the German Bundesliga was not influenced by the COVID-19 lockdown, contrary to those of the Polish Ekstraklasa. This difference could have been caused by different break lengths and different restrictions implemented in these countries during the pandemic lockdown.

## 1. Introduction

A novel type of coronavirus (2019-nCoV, SARS-CoV-2) infecting humans appeared in Wuhan, China, at the end of December 2019 [1]. The COVID-19 disease caused by this virus began to spread all over the world. Emerging publications have suggested that SARS-CoV-2 may negatively influence the immune system [2] and organs, such as the lungs [3], heart [4], and kidneys [5]. Therefore, subsequent countries decided to implement numerous restrictions to protect their citizens. These limitations clearly affected sport competition, including soccer. On 11 March 2020, the World Health Organization (WHO) officially declared COVID-19 a pandemic, and most national soccer federations decided to suspend all league games.

This lockdown concerned official matches as well as typical (team) training sessions in many countries. In countries such as Germany and Poland, from midway through March to the beginning of May, soccer teams were not allowed to perform the traditional training process. Moreover, until the end of April, coaches had no knowledge as to when they would be able to return to training routines and games. Therefore, in the middle of competition, an off-season break of unknown length occurred. The dilemma of how to divide this time into detraining and precompetition periods was a task for team staff.

Previously, scientific evidence established that detraining periods of short and long durations could negatively influence maximal oxygen uptake, sprint ability, muscle power, and players’ body composition [6]. Specifically, Koundourakis et al. [7] demonstrated that six weeks of detraining could significantly decrease maximal oxygen uptake (VO_2_max), jumping ability (squat jump—SJ, countermovement jump—CMJ), and speed ability in professional soccer players. Thus, the basic goal of coaches during the COVID-19 lockdown was to maintain the appropriate level of players’ physical fitness to enable sufficient match performance.

In many countries, soccer federations with medical committees prepared recommendations to support soccer teams before training and match resumption [8,9,10,11]. Most of this advice considers two basic issues: (1) How can potential infections be prevented? (2) How can infected persons proceed when they are confirmed? Carefully implemented recommendations involved social distancing, wearing masks, disinfecting hands, and systematically performing COVID-19 tests (antibody detection or real-time PCR tests). All these efforts allowed the season to be completed. German Bundesliga and Polish Ekstraklasa were among the first leagues to return to play. The first official matches after the lockdown were played by German Bundesliga after a 66-day break. In turn, Polish Ekstraklasa resumed competition 2 weeks later, on 29 May, after 81 days.

Physical performance during soccer matches is dependent on variables such as player age [12], ball possession [13], game location [14], match outcome [15], quality of opponent [16], and player body composition [17]. Moreover, the level of physical fitness is a factor that influences running performance during a game. The significant relationships between aerobic capacity and high-intensity running during games were previously shown [17].

High-intensity actions are crucial elements, especially in the most important moments of a game and for better understanding should be analyzed in relation to key tactical activities [18]. The tendencies in modern soccer, presented by Barnes et al. [19], clearly underline the importance of such physical variables as high-intensity running and sprinting. Furthermore, Konefał et al. [20] presented a predictive model that suggests that the importance of high-intensity running and sprinting may even increase in subsequent seasons. According to Guerrero-Calderon [21], the off-season period which occurred due to pandemic lockdown was expected to negatively influence the high-intensity performance of professional soccer players.

Recently, Brito de Souza et al. [22] emphasized the necessity of investigating the effect of the COVID-19 lockdown on performance in professional football players from different leagues. To the authors’ best knowledge, only one study previously evaluated the impact of pandemic season stoppage on physical match activity in professional soccer [23]. Two of the first professional soccer leagues that resumed competition were German Bundesliga and Polish Ekstraklasa. Therefore, the aim of the current study was to investigate whether the in-season pandemic lockdown influenced physical match performance in professional soccer players who participated in the competitions of German Bundesliga and Polish Ekstraklasa. We hypothesized that running performance after this extraordinary off-season period would be significantly reduced when compared to that in games played before the COVID-19 lockdown.

## 2. Materials and Methods

### 2.1. Study Design

Our observational retrospective study concerned the changes in the physical match performance of German Bundesliga and Polish Ekstraklasa players before and after the pandemic lockdown. A Bundesliga model contained 34 matchdays (18 teams play twice a season against each other), and the model of the Ekstraklasa season included 37 matchdays (ESA 37 model). This ESA 37 system consists of performing 30 games in the basic part of the season (16 teams play against each other twice). Afterwards, the league table is split into two subgroups of 8 teams (Champion group and Relegation group), at which point another 7 matches are played.

Each team from Bundesliga performed 25 games before and 9 games after the lockdown. In Poland, the pandemic occurred after completing 26 games, and 11 matches were played after resumption of the season. Different models of competition performed in Germany and Poland made the between-league comparison problematic. Moreover, different tracking systems used in the analyzed leagues and different divisions of speed zones did not allow for reliable comparisons between Bundesliga and Ekstraklasa.

This study maintained the anonymity of the players following data protection law. The study was conducted in accordance with the Declaration of Helsinki and approved by the Local Research Ethical Committee (agreement number: 12/2021).

### 2.2. German Bundesliga Schedule

**Figure 1:**
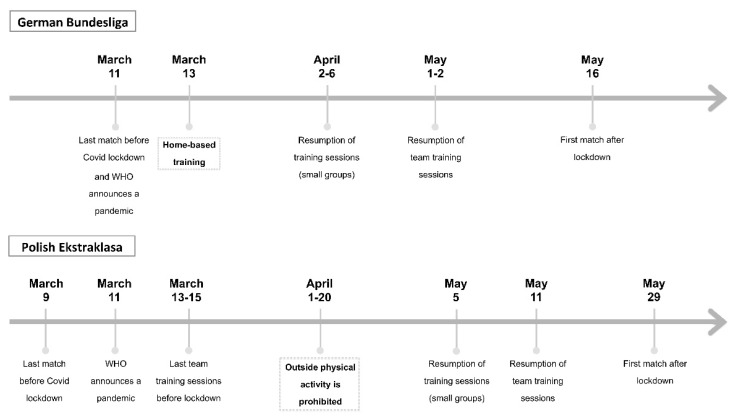
Time schedule involving important dates of COVID-19 lockdown in Germany and Poland.

The opening game of the 2019/2020 German Bundesliga season was played between Bayern Munich and Hertha Berlin on 18 August. The last match before the COVID-19 lockdown was played on 11 March. On the same day, the World Health Organization (WHO) announced COVID-19 as a worldwide pandemic. After suspension of the competition, players received individual programmes for home-based training, which was performed between 13 March and 1 April. This training usually involved exercises improving aerobic capacity (running or working on the cycloergometer) and strength training with the use of body weights. These sessions were performed in most of the clubs 5–6 times a week. After that date, Bundesliga teams were allowed to perform soccer training in small groups. The regular team training sessions were implemented at the beginning of May. Two weeks later (on 16 May), the first post-lockdown matches were played. Bundesliga was the first professional soccer league to resume competition after the COVID-19 break. The final part of the season that included 9 games was completed on 27 June (Figure 1).

### 2.3. Polish Ekstraklasa Schedule

The season schedule of Polish Ekstraklasa established the first games on 19 July and final matches on 17 May. Due to the pandemic, the season was extended by another nine weeks. On 9 March, the last game before the lockdown was played. From half-way through March to the 5th of May, no group training sessions were played. During this period of time, players trained individually outside or in their homes. Similar to Bundesliga, these sessions were performed 5–6 times a week and usually involved developing aerobic fitness and resistance (strength) training. The largest restrictions were enforced by Polish authorities in April, when physical activities were prohibited even outside (in the parks or forest). At the beginning of May, after more than seven weeks, players resumed their training in small groups (6–10 players; 60–75 min) during which large number of soccer-specific drills were performed. Team training sessions restarted on 11 May, which was eighteen days before competition resumption. None of the teams were allowed to play friendly games at this time. All these complications provided numerous difficulties for coaches and sport scientists in preparing players for the final part of the season. Detailed information about the lockdown in Germany and Poland is presented in Figure 1.

### 2.4. Methodology

#### 2.4.1. German Bundesliga

In total, 306 Bundesliga matches were included in the research (225 before and 81 after the COVID-19 lockdown). The analysis of match physical activities in German Bundesliga was carried out with the use of VIS. TRACK provided by IMPIRE AG (Ismaning, Germany) and Cairos Technologies AG (Karlsbad, Germany). This previously described [24,25] tracking system with a sampling frequency of 25 Hz provides the data of variables, such as total distance (TD) and distance covered in different speed zones (11–14 km/h—low-intensity running; 14–17 km/h—moderate intensity running; 17–21 km/h—high-intensity running; 21–24 km/h—very high-intensity running; >24 km/h—sprinting). All physical performance variables were calculated for total match duration and normalized to meters per minute (m/min). Moreover, the VIS. TRACK system supplies information on effective playing time (E_t_), which is defined as the duration of play after subtracting the time taken up by all stoppages—when balls are out of play (injuries, substitutions, VAR interventions) [14]. Additionally, the total distance in relation to effective playing time (TD (E_t_)) was calculated by dividing TD and effective playing time. Furthermore, the TD (E_t_) indicator is a quotient of the TD and E_t_.

#### 2.4.2. Polish Ekstraklasa

In total, 296 Polish Ekstraklasa matches (208 before and 88 after the pandemic break) from the 2019/2020 season were analyzed according to selected physical performance variables. Data on the physical performance in Polish Ekstraklasa were obtained from a previously validated [26] TRACAB optical video tracking system (ChyronHego, New York, NY, USA). TRACAB is a stereo camera system consisting of two multicamera units (each comprising three HD-SDI cameras with a resolution of 1920 × 1080 pixels) located on both sides of the half line. The frequency of sampling was 25 Hz (identical to that used in the Bundesliga VIS. TRACK system). Variables, such as total distance, low-intensity running (7.2–14.39 km/h), running (14.4–19.8 km/h), high-intensity running (HIR; 19.8–25.2 km/h), and sprinting (>25.2 km/h), were measured. The total number of high-intensity runs and sprints was defined as the number of high-intensity actions (HIAs). The total match duration and E_t_ data were also obtained from the TRACAB system. Thus, TD and distance covered in particular speed zones were calculated for both total and effective playing time and normalized per unit of time in the same way as the Bundesliga data.

### 2.5. Statistical Analysis

All analyses were conducted using the statistical software R version 3.6.3 (R Core Team, Auckland, New Zealand) [27]. The descriptive results for each variable are reported as the mean and standard deviations (SD). Linear mixed models were adjusted using the R package “lme4” [28] to analyze the differences between the team’s physical performance before and after the COVID-19 lockdown (fixed factor). Team identity was modelled as a random effect to account for the repeated measurements. The assumption of homogeneity and normal distribution of the residuals was established for each model. All models were normally distributed and displayed homogenous variance. The percentage of change for each variable was calculated ((After − Before/Before) × 100). Additionally, effect sizes (ES) were determined using Cohen’s *d*. According to Cohen [29], effect sizes (ES) were classified as trivial (*d* < 0.2), small (0.2 ≤ *d* < 0.5), medium (0.5 ≤ *d* < 0.8), and large (*d* > 0.8). Significance was established at the *p* ≤ 0.05 levels.

## 3. Results

### 3.1. German Bundesliga

**Figure 2:**
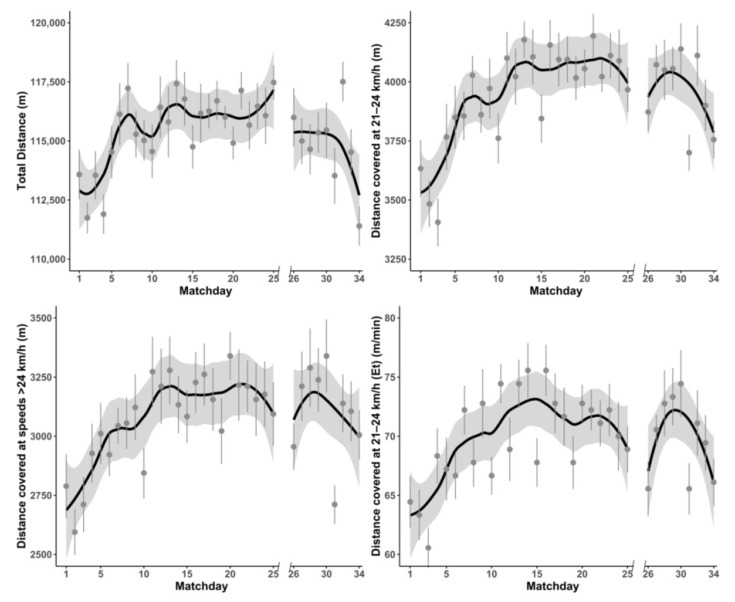
Changes in selected physical performance variables before and after the pandemic lockdown in German Bundesliga teams during official matches.

Table 1 shows the differences in match running performance before and after the COVID-19 lockdown in German Bundesliga. Figure 2 displays the effects of the COVID-19 lockdown on the trend in match running performance. There were no significant differences found in TD (*F* = 3.081; *p* = 0.08), distance covered at 21–23 km/h (*F* = 0.146; *p* = 0.702), or distance covered at >24 km/h (*F* = 1.661; *p* = 0.198). Regarding total playing time and effective playing, no significant differences were revealed for any relative match running performance variable before and after the COVID-19 lockdown. In addition, no significant differences were found in E_t_ (*F* = 0.357; *p* = 0.550) before and after COVID-19 lockdown.

### 3.2. Polish Ekstraklasa

**Figure 3:**
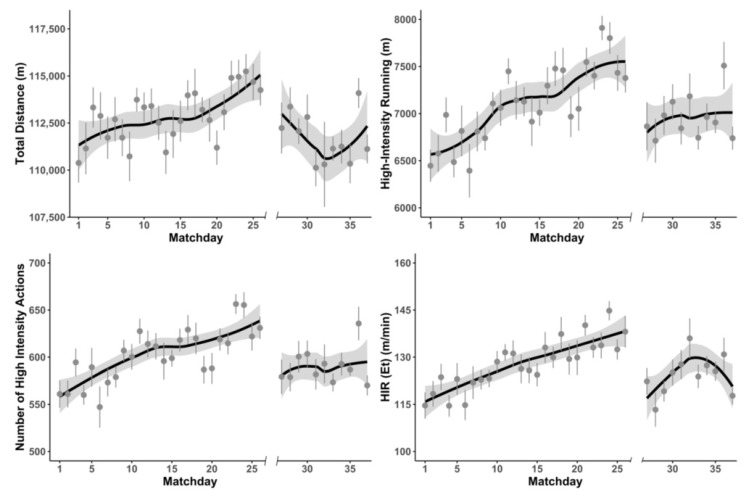
Changes in selected physical performance variables before and after the pandemic lockdown in German Bundesliga teams during official matches (HIR—high-intensity running, E_t_—effective playing time).

Table 2 presents the differences in match running performance before and after the COVID-19 lockdown in Polish Ekstraklasa. Figure 3 displays the effects of the COVID-19 lockdown on the trend in match running performance. The linear mixed model showed that teams covered significantly lower TD (*F* = 12.108; *p* < 0.001), HIR distance (*F* = 4.586; *p* = 0.03), and number of HIAs (*F* = 5.492; *p* = 0.02) after the COVID-19 lockdown. Moreover, taking into account total playing time, no significant differences were found for any variable of relative match running performance before and after the COVID-19 lockdown. When effective playing time was considered, teams covered significantly less relative TD (Et) (*F* = 7.171; *p* < 0.001) and HIR (Et) distance (*F* = 5.479; *p* = 0.02). However, no significant differences were found in E_t_ (*F* = 0.784; *p* = 0.38) before and after lockdown performance.

## 4. Discussion

The main purpose of this study was to evaluate the physical match performance before and after the COVID-19 lockdown in professional soccer players from two professional European leagues, the German Bundesliga and Polish Ekstraklasa. The main finding of our research is that the physical performance of German Bundesliga teams was less influenced by the COVID-19 lockdown than the performance of Polish Ekstraklasa teams. Significant decreases in variables, such as total distance, distance covered in high-intensity running, number of high-intensity actions, and relative total distance in relation to effective playing time, were reported only in Polish Ekstraklasa. However, the data presented in Figure 2 may suggest some reduction in Bundesliga team physical performance during the final part of the season.

The most recent research of Garcia-Aliaga et al. [23] compared the physical match activity during two blocks of 11 matches (first 11 games of the season 2019/2020, and 11 games after the COVID-19 lockdown) in Spanish LaLiga^TM^. The significant decrease in such performance indicators as distance, high-speed running, sprinting, and number of high-intensity actions were reported. Moreover, the relative values of some of these indicators were lower after the season resumption as well. Interestingly, the number of accelerations and decelerations per minute was higher during the post-lockdown phase. Pandemic lockdown for professional soccer players in Spain lasted 12 weeks (8 weeks of home-based training followed by 20 days of individual soccer training and 14 days of typical group sessions). During this period, most of the training sessions were not performed in soccer-specific context, which could affect the training intensity.

Differences between physical performances immediately after the lockdown in Bundesliga and Ekstraklasa could have been caused by the length of the lockdown and training possibility during this break. Brito de Souza et al. [22] analyzed the changes in Spanish LaLiga players’ physical performance among the last four seasons and suggested that, after the usual 12-week summer break, players need approximately 8–10 matches to reach match running performance stabilization. Data from the beginning of the season in both Bundesliga and Ekstraklasa seem to confirm this statement (Figure 2 and Figure 3). The pandemic break between the matches in Poland was longer, lasting 81 days, which is very close to the 12 weeks mentioned, while German Bundesliga resumed competition after approximately 9 weeks. Moreover, in Germany, soccer players restarted the training process in small groups 40–44 days before season resumption, while in Poland, players were allowed to participate in small group training sessions 24 days and in full team sessions only 18 days before the first post-lockdown game. This short period of preparation seemed to be insufficient for the effective preparation of professional soccer players.

Similarly, a lower level of performance in most physical variables at the beginning of the season was reported by Chmura et al. [30], who analyzed the physical and technical performance of Bundesliga players among three consecutive seasons. Furthermore, they found a statistically significant decline in some physical variables in the last phase of the season (last five matches). Data presented in the current study show similar drops in German Bundesliga at the final games (Figure 2). However, this tendency was not observed in Polish Ekstraklasa matches.

In the opinion of international soccer experts, running at maximum and sub-maximum speeds is one of the variables which is both the most important, and the most affected by coronavirus quarantine [21]. Thus, the decreased distance at HIR and number of HIAs performed by Ekstraklasa players after the lockdown were somehow expected. In contrast, the Bundesliga teams were able to avoid such drops. The lower level of high-intensity activities performed by the Polish Ekstraklasa team after the lockdown could probably be explained by the longer period of time without training sessions than in German Bundesliga.

Reduced match performance could be determined by changes in physical fitness and body composition [17]. Grazioli et al. [31] compared the changes in neuromuscular, body composition, and cardiorespiratory parameters during traditional off-season breaks (24 days) and COVID-19 quarantine (63 days) in Brazilian professional soccer players. The significantly lower results of countermovement jumps at height and significantly longer times in the 10 m and 20 m sprints suggest that longer breaks particularly affect the dynamic components of physical fitness. Moreover, the body mass and fat mass of these players significantly increased. Furthermore, the results presented in the paper of Korkmaz et al. [32] showed that, during 89 days of lockdown, significant reductions in anaerobic power and body muscle mass were observed in semi-professional soccer players. According to all these data, special attention should be given to body composition-related and speed power-related capabilities after long-term detraining in professional soccer. Additionally, the comparison of physical capacity and body composition test results performed before and after the lockdown could provide interesting conclusions and answer the question of whether reduced match performance was connected with the decline in physical fitness.

Several international institutions and sport scientists introduced recommendations about returning to training and competition [9,10,11,33]. Concretely, Mohr et al. [34] stated that, due to the lack of matches and soccer-specific intense actions, lowered soccer-related physical fitness is expected after the COVID-19 lockdown. Moreover, these authors provided basic principles for progressively developing physical fitness components, such as aerobic and anaerobic capacity or power. Additionally, the importance of injury prevention programmes was also underlined. All these aspects should be taken into consideration when preparing players to resume the season. A rule modification that could potentially affect the match performance was applied after the COVID-19 lockdown. The Fédération Internationale de Football Association (FIFA) allowed coaches to make five substitutions during the game instead of the three previously available changes. The purpose of this decision was to reduce the potential risk of injuries. Previous studies [35,36] showed that substitute players cover 9–10% longer distances in HIRs than replaced players, and 24–27% longer distances than players who played the whole game. Lorenzo-Martinez et al. [37] showed that this improvement occurs independently of the playing position. Two more substituted players on the pitch should theoretically improve team physical performance. This could be one of the factors that helped German Bundesliga players maintain the previous level of physical performance after season resumption. However, the same rule was implemented in Polish Ekstraklasa, where significant decreases in TD and HIR were observed.

The authors are fully aware of numerous factors that could have influenced the results of the presented analysis, not including a number of contextual variables (e.g., home advantage, match outcome, strength of opponent, position on the pitch). Due to the large variety and lack of access to official data, the description of applied training loads during the COVID-19 lockdown in the Bundesliga and Ekstraklasa teams was not included. Moreover, different models of season and different tracking systems (dividing data into different speed zones) used in Bundesliga and Ekstraklasa did not allow for reliable comparison between the leagues. Finally, a larger number of matches, played with only 72 h long recovery gap, were played after resuming the season. Future research on the influence of COVID-19 lockdown on injury incidence and physical and technical activities in other professional leagues, where different lengths of lockdown occurred, may be an interesting issue. The analysis of individual changes according to playing position should also be deeply investigated in further studies.

## 5. Conclusions

Only one study [23] has previously investigated the influence of the COVID-19 lockdown on the physical performance of professional soccer players. The extraordinary period without playing friendly games resulted in limitations in the level of soccer-specific physical fitness, including the ability to perform high-intensity activities. However, the duration of the lockdown, implemented restrictions, physical potential of the players, and training loads applied during the COVID-19 break differentiate the match performance after the season resumption in analyzed leagues. Certain variables, such as distance covered in high-intensity running and the number of high-intensity activities, were particularly affected in Polish Ekstraklasa, while no significant changes in any of the physical performance variables were observed in German Bundesliga teams. German Bundesliga and Polish Ekstraklasa were two of the first competitive leagues that returned to play after the COVID-19 lockdown. Therefore, studies considering the data from these leagues provide practical information that can be effectively used while planning physical loads for players after long-term training absence. Players with high levels of aerobic and anaerobic capacity better abide a long-term training break. Thus, applying exercises which promote a large number of high-intensity efforts (e.g., small-sided games or exercises involving a large number of HIRs) can prevent a decline in the locomotion activities.

## Figures and Tables

**Table 1:** Changes of physical and technical match performance in German League before and after the COVID-19 breakdown (mean and SD).

	Before	After	∆ (%)	ES (CI 95%)
Total Distance (m)	115,501.1 ± 4444.2	114,822.2 ± 4530.9	−0.59	−0.15 (−0.33 to 0.03)
Distance covered at 21–24 km/h (m)	3946.7 ± 480.1	3961.7 ± 442.6	0.38	0.03 (−0.14 to 0.21)
Distance covered at speeds >24 km/h (m)	3074.4 ± 555.0	3110.5 ± 564.2	1.17	0.06 (−0.11 to 0.24)
E_t_ (min)	56.8 ± 4.2	57.0 ± 4.8	0.35	−0.05 (−0.13 to 0.22)
TD (m/min)	1213.42 ± 49.05	1207.68 ± 49.20	−0.46	−0.12 (−0.30 to 0.06)
TD (E_t_) (m/min)	2042.3 ± 134.3	2025.2 ± 149.5	−0.84	−0.12 (−0.30 to 0.06)
Distance covered at 21–24 km/h (m/min)	41.48 ± 5.20	41.68 ± 4.80	0.48	0.04 (−0.14 to 0.22)
Distance covered at 21–24 km/h (E_t_) (m/min)	69.8 ± 9.8	69.9 ± 10.3	0.14	0.01 (−0.16 to 0.19)
Distance covered at speeds >24 km/h (m/min)	32.32 ± 5.96	32.73 ± 6.02	1.27	0.07 (−0.11 to 0.25)
Distance covered at speeds >24 km/h (E_t_) (m/min)	54.2 ± 10.7	55.3 ± 11.6	2.03	0.10 (−0.08 to 0.28)

**Table 2:** Changes of physical match performance before and after the COVID-19 lockdown in Polish Ekstraklasa.

	Before	After	∆ (%)	ES (95% CI)
TD (m)	112,895.0 ± 4218.6	111,714.4 ± 4614.1 **	−1.05	−0.27 (−0.45 to −0.10)
HIR (m)	7107.3 ± 838.5	6961.2 ± 786.6 *	−2.06	−0.18 (−0.35 to −0.01)
Sprint (m)	1752.9 ± 357.7	1720.6 ± 332.6	−1.84	−0.09 (−0.27 to −0.01)
Number of HIR	602.3 ± 62.5	590.5 ± 60.3 *	−1.96	−0.20 (−0.37 to −0.02)
E_t_ (min)	55.9 ± 4.7	56.3 ± 4.6	0.71	0.09 (−0.09 to 0.26)
TD (m/min)	1166.1 ± 49.3	1162.3 ± 50.8	−0.33	−0.08 (−0.25 to 0.10)
TD (E_t_) (m/min)	2030.1 ± 159.8	1995.3 ± 162.3 *	−1.72	−0.21 (−0.39 to −0.04)
HIR (m/min)	73.53 ± 8.59	72.43 ± 8.34	−1.50	−0.13 (−0.31 to 0.05)
HIR (E_t_) (m/min)	127.95 ± 17.86	124.45 ± 17.78 *	−2.74	−0.20 (−0.37 to −0.02)
Sprint (m/min)	18.13 ± 3.65	17.90 ± 3.48	−1.27	−0.06 (−0.24 to 0.11)
Sprint (E_t_) (m/min)	31.57 ± 6.94	30.71 ± 6.41	−2.72	−0.13 (−0.30 to 0.05)

* Significant difference between Before and After (*p* < 0.01); ** Significant difference between Before and After (*p* < 0.05). ES—effect size, CI—confidence interval, TD—total distance, HIR—high speed running, HIA—high-intensity actions, and E_t_—effective playing time.

## Data Availability

The data used for this study was acquired from a third-party, https://matchanalysishub.bundesliga.com/login (access on 1 December 2020) and https://tracabportal.azurewebsites.net/login (access on 1 April 2021). The data was provided under scientific cooperation with a football clubs currently appearing in Bundesliga and Ekstraklasa. The authors’ ethical approval also prevents them from sharing any data in any way that could be re-identified. The metadata would allow someone else to re-identify teams and possibly players. However, access to the data should be possible from the third-party. The data acquired were so called ‘excel dumps’ of player statistics per match. Access to the data can be organized by contacting Match Analysis Hub: mdc@sportec-solutions.de and info@chyronhego.com.
